# Molecular characterization and three-dimensional structures of avian H8, H11, H14, H15 and swine H4 influenza virus hemagglutinins

**DOI:** 10.1016/j.heliyon.2020.e04068

**Published:** 2020-06-06

**Authors:** Hua Yang, Paul J. Carney, Jessie C. Chang, James Stevens

**Affiliations:** Influenza Division, National Center for Immunization and Respiratory Diseases, Centers for Disease Control and Prevention, Atlanta, GA 30329, USA

**Keywords:** Microbiology, Virology, Viral protein, Proteins, Biomolecules, Glycobiology, Hemagglutinin, Influenza virus, Avian, Swine, Receptor binding, A(H8N4), A(H11N9), A(H14N5), A(H15N9), A(H4N6)

## Abstract

Of the eighteen hemagglutinin (HA) subtypes (H1–H18) that have been identified in bats and aquatic birds, many HA subtypes have been structurally characterized. However, several subtypes (H8, H11 and H12) still require characterization. To better understand all of these HA subtypes at the molecular level, HA structures from an A(H4N6) (A/swine/Missouri/A01727926/2015), an A(H8N4) (A/turkey/Ontario/6118/1968), an A(H11N9) (A/duck/Memphis/546/1974), an A(H14N5) A/mallard/Gurjev/263/1982, and an A(H15N9) (A/wedge-tailed shearwater/Western Australia/2576/1979 were determined by X-ray crystallography at 2.2Å, 2.3Å, 2.8Å, 3.0Å and 2.5Å resolution, respectively. The interactions between these viruses and host receptors were studied utilizing glycan-binding analyses with their recombinant HA. The data show that all avian HAs retain their strict binding preference to avian receptors, whereas swine H4 has a weak human receptor binding. The molecular characterization and structural analyses of the HA from these zoonotic influenza viruses not only provide a deeper appreciation and understanding of the structure of all HA subtypes, but also re-iterate why continuous global surveillance is needed.

## 1 Introduction

Influenza is an acute respiratory illness, caused by influenza A, B, C and D viruses (Hause et al., 2014; Palese and Shaw, 2007). While all of these viruses contain segmented, linear, negative-sense, single-stranded RNA genomes (Fields et al., 2007), they differ in the number of RNA segments, with eight for influenza A and B and seven for influenza C and D. Influenza A viruses (IAVs) are the most prevalent pathogen for both humans and animals (Cox and Subbarao, 2000). Based on the influenza virus' antigenic surface glycoproteins, sixteen hemagglutinin (HA) (H1–H16) and nine neuraminidase (NA) (N1–N9) circulate in aquatic birds (Palese and Shaw, 2007), and two subtypes, A(H17N10) and A(H18N11) have been identified from bats (Tong et al., 2012, 2013). In birds alone, there could be as many as 144 possible HA/NA combinations. However, many HA/NA combinations have yet to be detected (Wille et al., 2018). While H3, H4 and H6 avian influenza virus (AIV) subtypes are common, H8–H12, H14 and H15 are rarely detected in wild aquatic birds, while H13 and H16 viruses have been isolated mainly from gulls (Wille et al., 2011).

HA and NA both play an important role during the virus life cycle. Influenza virus infection is initiated by HA binding to sialic acid receptors and mediates virus entry and fusion (Skehel and Wiley, 2000), while NA cleaves sialic acid from the infected host cell, allowing release of progeny viruses. The HAs of human influenza viruses preferentially bind to glycan receptors with terminal α2-6 linked sialic acid, whereas the HAs of avian IAVs bind to receptors with α2-3 linked sialic acid (Matrosovich et al., 2000; Rogers et al., 1985). Although interspecies transmission of influenza viruses between avian and human hosts is rare, subtypes such as A(H5N1), A(H5N6), A(H6N1), A(H7N2), A(H7N3), A(H7N4), A(H7N7), A(H7N9), A(H9N2), A(H10N7), A(H10N8) have crossed the species barrier and caused sporadic human infections and death (Chen et al., 2014; Fouchier et al., 2004; Parry, 2013; Peiris et al., 1999; Shi et al., 2013; To et al., 2014; WHO, 2018; Wong and Yuen, 2006; Yuen et al., 1998).

Previous studies identified a number of key receptor binding site (RBS) mutations of HA, responsible for switching avian/human receptor specificity in H1, H2 and H3 subtypes. In H1 subtypes, a Glu190Asp and Gly225Asp double mutation renders the HA capable of binding human α2-6 receptors (Stevens et al., 2006). For H2 and H3, two different mutations, Gln226Leu and Gly228Ser correlate with a shift to human receptor specificity (Connor et al., 1994; Rogers et al., 1983).

**Figure 1:**
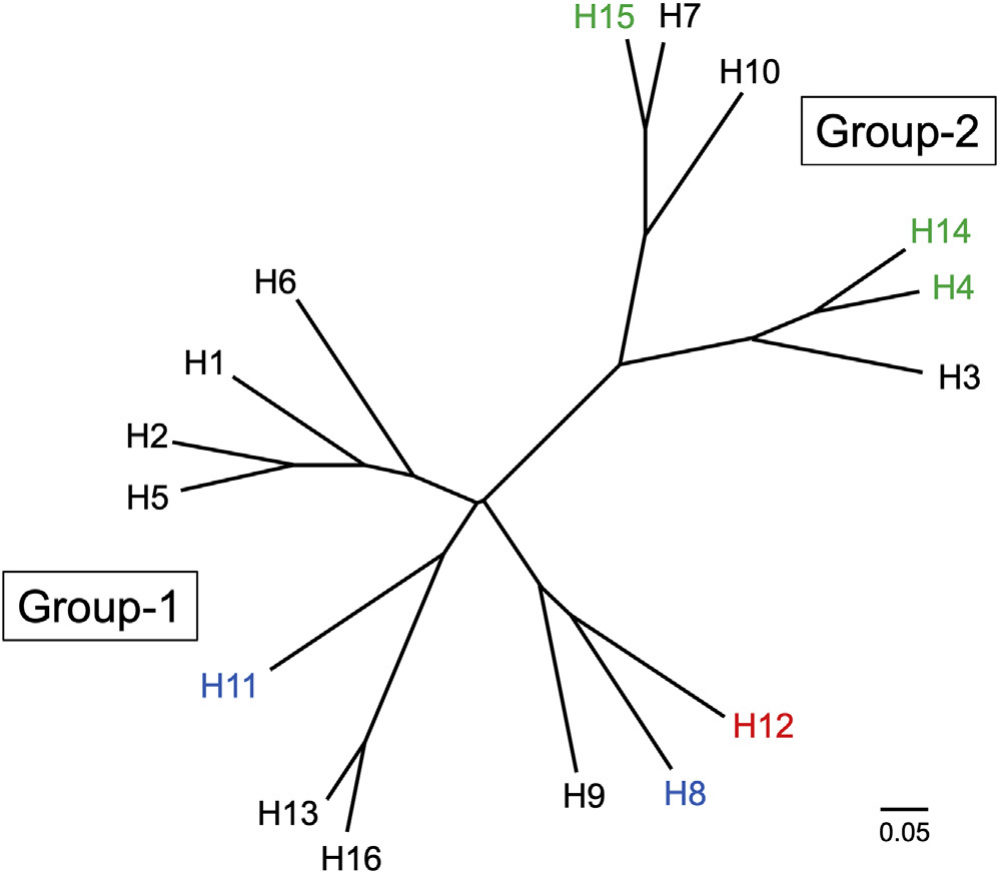
Influenza A virus HA phylogenetic tree. The HAs can be divided into group-1 and group-2, which can both be subdivided into subgroups. The discussed structures of H8 and H11 in group 1 are highlighted in blue, while H4, H14 and H15 in group 2, are highlighted in green. H12 HA, which is colored in red, is the only HA not in the Protein Data Bank (PDB).

Phylogenetic analysis reveals that all HA subtypes can be separated into two groups, and each group further divided into subgroups (Figure 1) (Gamblin and Skehel, 2010).

Indeed, almost four decades have elapsed since the first crystal structure of influenza virus HA was determined and it facilitated an understanding of the structural identification of the major antigenic sites and the effects of natural variation (Wilson et al., 1981). Among all HA subtypes, H8, H11 and H12 HAs have yet to be structurally characterized. In this study, we focus on molecular characterization of HAs from an A(H8N4) (A/turkey/Ontario/6118/1968), an A(H11N9) (A/duck/Memphis/546/1974), an A(H14N5) A/mallard/Gurjev/263/1982, an A(H15N9) (A/wedge-tailed shearwater/Western Australia/2576/1979, and an A(H4N6) A/swine/Missouri/A01727926/2015) (Table 1).

**Table 1:** Recombinant HA proteins used in this study.

Strain (Subtype)	Accession Number		Abbreviation
	GISAID	NCBI	
A/swine/Missouri/A01727926/2015 (H4N6)	EPI_ISL_213836	AMK09582	swH4
A/turkey/Ontario/6118/1968 (H8N4)	EPI_ISL_70124	ABI84519	avH8
A/duck/Memphis/546/1974 (H11N9)	EPI_ISL_69885	ABI84556	avH11
A/mallard/Gurjev/263/1982 (H14N5)	EPI_ISL_14744	GQ247868	avH14
A/wedge-tailed shearwater/Western Australia/2576/1979 (H15N9)	EPI_ISL_8917	ABB88138	avH15
A/Switzerland/9715293/2013	EPI814528	–	HuH3
A/Vietnam/1203/2004	EPI361524	AAW80717	AvH5

## 2 Results

### 2.1 Group-1 avH8 and avH11 AIV HAs

**Figure 2:**
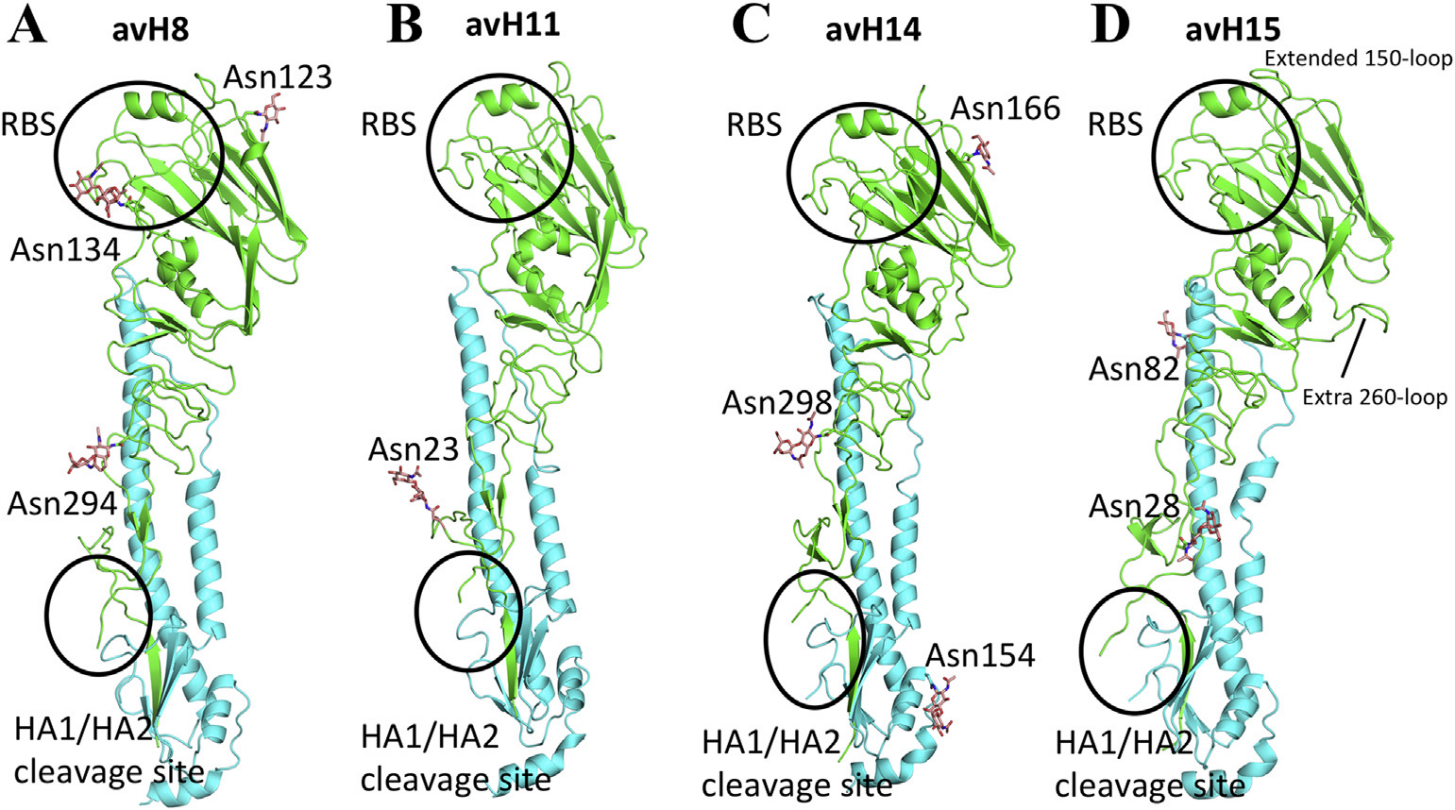
The overall structure of HA monomer. A. The overall structure of group-1 avH8 monomer, with occupied glycosylation sites shown as sticks. B. The overall structure of group-1 avH11 monomer with occupied glycosylation sites shown as sticks. C. The overall structure of avH14 monomer with occupied glycosylation sites shown as sticks. D. The overall structure of avH15 monomer with occupied glycosylation sites shown as sticks.

**Figure 3:**
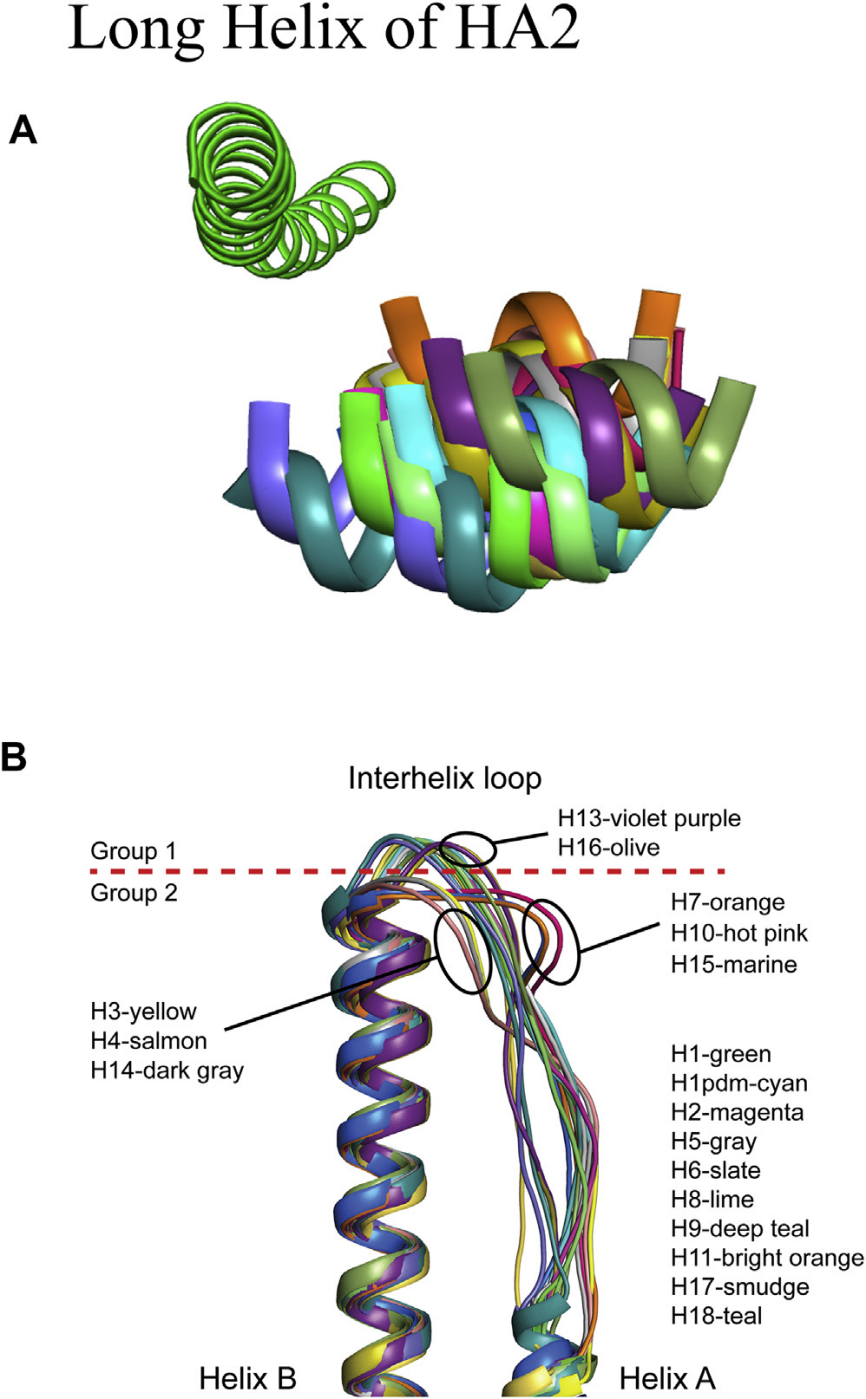
Superimpositions of all available HAs. A. The rotation of head domain is represented by the 190-helix in the RBS of each HA subtype. The long α helix of the HA2 was used to perform superimposition of different subtype HAs. B. The overlap of interhelix. The PDBs used in the alignment are: H1_1RD8, H1pdm_3M6S, H2_2WRC, H3_4WE4, H5_2FK0, H6_4WST, H7_6D7C, H9_1JSD, H10_4WSX, avH11, H13-4KPQ, avH14, avH15, H16_4F23, H17_4I78, H18_4K3X.

Many subtypes of HAs have been structurally determined previously, but H8 and H11 HAs are not represented in the Protein Data Bank (PDB). Both H8 and H11 are group-1 HAs (Figure 1), but they reside in different subgroups. H8 HA groups with H9 and H12 HAs, while H11 HA groups with H13 and H16 HAs. We chose to study two AIVs by: an A(H8N4) (A/turkey/Ontario/6118/1968), and an A(H11N9) (A/duck/Memphis/546/1974). In vivo, viral infection occurs when the single-chain precursor viral HA protein (HA0) is cleaved by host proteases into the infectious HA1/HA2 trimeric form. In these baculovirus expression studies, although all HA proteins were produced in the HA0 form, subsequent digestion with trypsin to remove the trimerization tag, also cleaved the HA into the biologically active HA1/HA2 form. The avH8 HA structure was determined at 2.3 Å (Table 2). As expected, the overall avH8 HA structure is very similar to other HAs (Figure 2A). Seven predicted glycosylation sites are present at Asn10/Asn11, Asn123, Asn134, Asn206, Asn287, Asn294, and Asn481. Only Asn123, Asn134 and Asn294 on the HA1 had interpretable density for one or two *N*-acetylglucosamines (GlcNAc) (Figure 2A). With Cα atoms superimposed, the root mean square deviations (RMSD) are shown in Table 3. The avH8 had an RMSD of 1.28Å compared with its subgroup H9 HA (PDB ID: 1JSD). Previous studies of HA structures have shown some group-specific features at the interhelix loop and the rigid-body orientation of the globular domain (Lu et al., 2013). Here we performed superimpositions of the long α helix of the HA2 to reveal any displacements of the HA1 globular domain in all the available HA subtype structures. The rigid-body orientation of the globular domain is presented by the 190-helix in the RBS of each HA subtype (Figure 3A). Different degrees of rotations were observed and calculated among all HA subtypes, and while there was distinct difference between group-1 and group-2 HAs, there was no obvious subgroup clustering identified (Table 4). However, the conformation of the interhelix-loop showed significant clustering. The loop conformations for group-1 H13 and H16 HAs are positioned differently from other group-1 HAs, while the loop conformations for the group-2 H3, H4 and H14 HAs are different from H7, H10 and H15 (Figure 3B). The interhelix-loop undergoes extensive conformational changes during HA activation (Xu and Wilson, 2011); therefore the structural differences among different subtypes may suggest different mechanisms during this process.

**Table 2:** Crystallization, data collection and refinement statistics for the HA crystal structures.

	swH4	avH8	avH11	avH14	avH15
**Experimental**					
Protein Conc. (mg/ml)	14	13	15	17	14
Crystallization conditions	0.1M Tris-HCl, pH8.5, 30% (w/v) PEG 1000	0.05M Magnesium Chloride, 0.1M HEPES:NaOH pH7.5, 30% (v/v) PEG 550 MME	0.1M MOPS, pH7.0, 18% (w/v) PEG 4000	0.2M Sodium cacodylate trihydrate pH5.5, 20% (w/v) PEG4000	0.1M Sodium acetate trihydrate, pH5.0, 20% (v/v) PEG 4000
Cryoprotectant	None	None	20% glycerol	20% glycerol	None

**Data collection**					

Beamline collected	APS, 22-ID	APS, 22-ID	APS, 22-ID	APS, 22-BM	APS, 22-ID
Space group	P2_1_	C2	P2_1_2_1_2_1_	C2	C2
Cell dimensions (Å)	68.97, 240.01, 68.95	170.05, 98.09, 132.74	80.12, 121.15, 217.73	174.67,100.97, 236.08	108.67, 101.38, 163.27
Cell angle (º)	90, 119.86, 90	90.00, 115.20, 90.00	90, 90, 90	90.00, 103.93, 90.00	90, 90.74, 90
Resolution (Å)	50–2.20 (2.26–2.20)^a^	50–2.25 (2.33–2.25)	50–2.8 (2.90–2.80)	50–3.0 (3.11–3.0)	50–2.5 (2.59–2.5)
Total reflections	96492 (8968)	94690 (8943)	52133 (4302)	82593 (7598)	63339 (5739)
Unique reflections	95270 (8892)	91870 (8031)	47619 (4051)	79186 (7388)	61139 (5600)
Rsym	0.087(0.47)	0.085 (0.77)	0.12 (0.55)	0.116 (0.56)	0.069 (0.61)
Rpim	0.059 (0.31)	0.069 (0.37)	0.044 (0.26)	0.104 (0.47)	0.036 (0.44)
I/sigma	29.5 (3.6)	15.2 (1.8)	25.8/5.6	7.7 (1.7)	21.8 (1.8)
Completeness (%)	98.0 (99.7)	98.2 (96.8)	93.1 (100)	99.9 (100)	99.9 (99.8)
Redundancy	3.3 (3.4)	3.8 (3.3)	8.1 (5.8)	3.8 (3.7)	3.8 (3.5)
CC_1/2_^b^	0.99 (0.74)	1 (1)	1 (0.99)	1 (1)	1 (1)

**Refinement**					

Resolution (Å)	50–2.20 (2.26–2.20)	50–2.25 (2.33–2.25)	50–2.8 (2.9–2.8)	50–3.0 (3.11–3.0)	50–2.5 (2.59–2.50)
No. reflections	95259 (8889)	91046 (8030)	47362 (4050)	79094 (7388)	61079 (5592)
No. reflections (test)	4767 (446)	4563 (383)	2370 (207)	3949 (355)	3098 (296)
Rwork/Rfree	20.3/24.4	18.7/22.5	23.4/27.8	22.4/26.0	23.0/26.5
No. of atoms	11706	11928	11712	23322	11781
B Values (Å^2^)	49.30	47.70	58.31	60.30	65.84
Wilson B value (Å^2^)	50.68	35.31	55.12	44.24	55.10
r.m.s.d.- bond length (Å)	0.010	0.012	0.011	0.009	0.011
r.m.s.d.- bond angle (º)	1.228	1.275	1.811	1.795	1.488

**MolProbity**^c^**scores**					

Favored (%)	94.3	96.1	94.7	92.6	93.1
Outliers (%)	0.6	0.3	0.3	0.6	1.0
**PDB Code**	6V44	6V46	6V47	6V48	6V49

a Numbers in parentheses refer to the highest resolution shell.

b CC_1/2_ Pearson correlation coefficient between two random half data sets.

c Reference (Davis et al., 2007).

**Table 3:** Comparison of RMSD (Å) for HA monomers.

	avH8	avH11	avH14	avH15	swH4
H1_1RD8	1.23	1.26	1.69	1.90	1.85
H1pdm_3M6S	1.22	1.18	1.63	1.75	1.69
H2_2WRC	1.29	1.23	1.56	1.55	1.63
H3_4WE4	1.73	1.77	0.68	1.20	0.70
H5_2FK0	1.47	1.24	1.40	1.53	1.39
H6_4WST	1.31	1.20	1.63	1.90	1.63
H7_6D7C	1.43	1.83	1.18	0.86	1.09
H9_1JSD	1.28	1.30	1.77	1.73	1.77
H10_WSX	1.79	1.79	1.25	0.93	1.30
H13_4KPQ	1.34	1.44	1.55	1.76	1.50
H16_4F23	1.43	1.55	1.66	1.61	1.67
H17_4I78	1.34	1.57	1.32	1.26	1.37
H18_4K3X	1.25	1.34	1.74	1.80	1.67

**Table 4:** The head rotation among different subtype HAs.

Group-1 (comparing to H1pdm)		Group-2 (comparing to H3) ∗	
H1	9.54^°^	H4	6.07^°^
H2	7.07^°^	H7	3.10^°^
H5	4.54^°^	H10	0.93^°^
H6	5.54^°^	H14	3.49^°^
H8	11.11^°^	H15	4.65^°^
H9	13.01^°^		
H11	8.64^°^		
H13	5.97^°^		
H16	13.19^°^		
H17	14.83^°^		
H18	7.82^°^		

∗ H1pdm vs H3 is 27.67^°^.

Among the rare AIVs, H11 has the highest number of entries in the GISAID database. A(H11) viruses have been isolated from different species of waterfowls that are located globally. The avH11 HA structure was determined at 2.8Å (Table 2): it shows no significant difference with other subtype HAs with RMSD all less than 2Å (Table 3). In comparison to the same subgroup containing H13 HA (PDB ID: 4KPQ) and H16 HA (PDB ID: 4F23), the RMSD values are 1.44Å and 1.55Å respectively. Among the five predicted glycosylation sites (Asn10/Asn11, Asn23, Asn165, Asn288, and Asn480), only Asn23 on HA1 has interpreted density for glycan (Figure 2B).

### 2.2 Group-2 avH14 and avH15 AIV HAs

For group-2 viruses, we chose an A(H14N5) A/mallard/Gurjev/263/1982 HA, and an A(H15N9) A/wedge-tailed shearwater/Western Australia/2576/1979 HA to assess their structural and binding properties. H14 and H15 are two AIV subtypes with only 45 and 22 sequences deposited in the GISAID database. Crystal structures of an H14N6 virus (PDB ID: 3EYJ) and H15N9 (PDB ID: 5TG8) HAs have been published before (Russell et al., 2008; Tzarum et al., 2017). The avH14 HA structure reported here was determined by X-ray crystallography to 3.0Å (Table 2), and it shows high similarity with other HAs, especially to its group-2 subgroup H3 HA with RMSD of 0.68Å (Table 3). Among seven predicted glycosylation sites (Asn5, Asn22, Asn46, Asn166, Asn226, Asn298 and Asn485), only Asn166 and Asn298 on HA1, and Asn154 on HA2 have interpreted density for the glycan (Figure 2C). The previous published H14 structure (PDB ID: 3EYJ) did not report any interpretable density for carbohydrates. The avH15 HA structure was determined at 2.5Å (Table 2) and it also resembles the other HAs with the highest similarity to its subgroup H7 HA. Among seven predicted glycosylation sites on avH15 HA (Asn12, Asn28, Asn82, Asn156, Asn413, Asn485 and Asn489), Asn28 on HA1 and Asn82 on HA2 have interpreted density for one or two *N*-acetylglucosamines (GlcNAc) (Figure 2D), consistent with the previously reported structure (Tzarum et al., 2017). Superimposition of their interhelix loops with other HA structures reveal that both H14 and H15 closely resemble and cluster with their subgroup HAs (Figure 3B). The structure of avH15 HA has a subgroup-specific feature as H7 and H10 HAs, whereby a two amino acid insertion, making an extended 150-loop, folds over towards the RBS and previous data indicates that the 150-loop may play a role in restricting the host specificity of A(H10N8) viruses (Tzarum et al., 2017). Moreover, one unique feature of H15 is the 7 to 10 amino acid insertion from 253-262, making an extra 260-loop (Figure 2D). In addition, the avH15 HA 260-loop results in a wider HA head creating a larger exposed surface that was proposed as contributing to increased antigenic variation in H15 HAs (Tzarum et al., 2017).

### 2.3 Receptor binding site analysis

**Figure 4:**
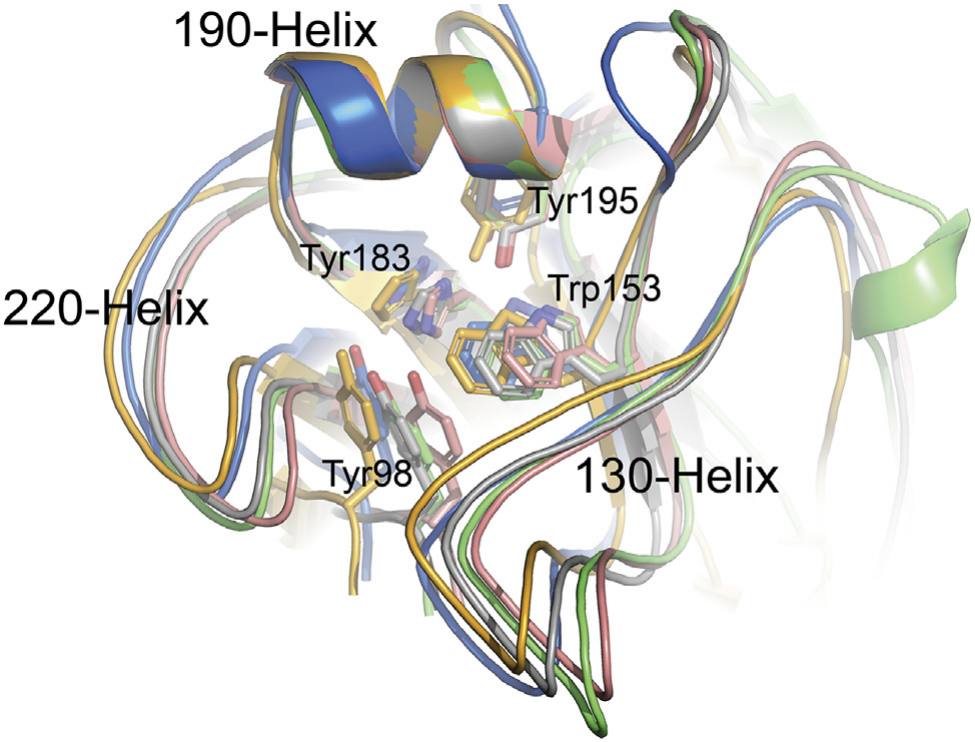
Superimposition of swH4, avH8, avH11, avH14 and avH15 RBS. The alignment was performed using the 190-helix. The three structural components and highly conserved RBS residues are labeled. The HA colors are consistent with those used in Figure 3.

Three HA receptor binding sites (RBS) are present in the HA trimer and are located at the top of each monomer. Including the 5 HAs discussed here, three major structural elements form the RBS binding pocket in all current HAs: a 190-helix (residues 188 to 194 based on H3 numbering), a 220-loop (residues 221 to 228), and a 130-loop (residues 134 to 138). In addition, highly conserved residues (Tyr98, Trp153, His183, and Tyr195) are also present in the base of the pocket of all 5 HAs and have been shown to affect receptor binding (Figure 4). When using 190-helix to align the RBS, the four avHAs and swHA show the same conformation of RBS with only slight variations in the 220-loop and 130-loop.

### 2.4 Glycan binding analyses of avHAs

**Figure 5:**
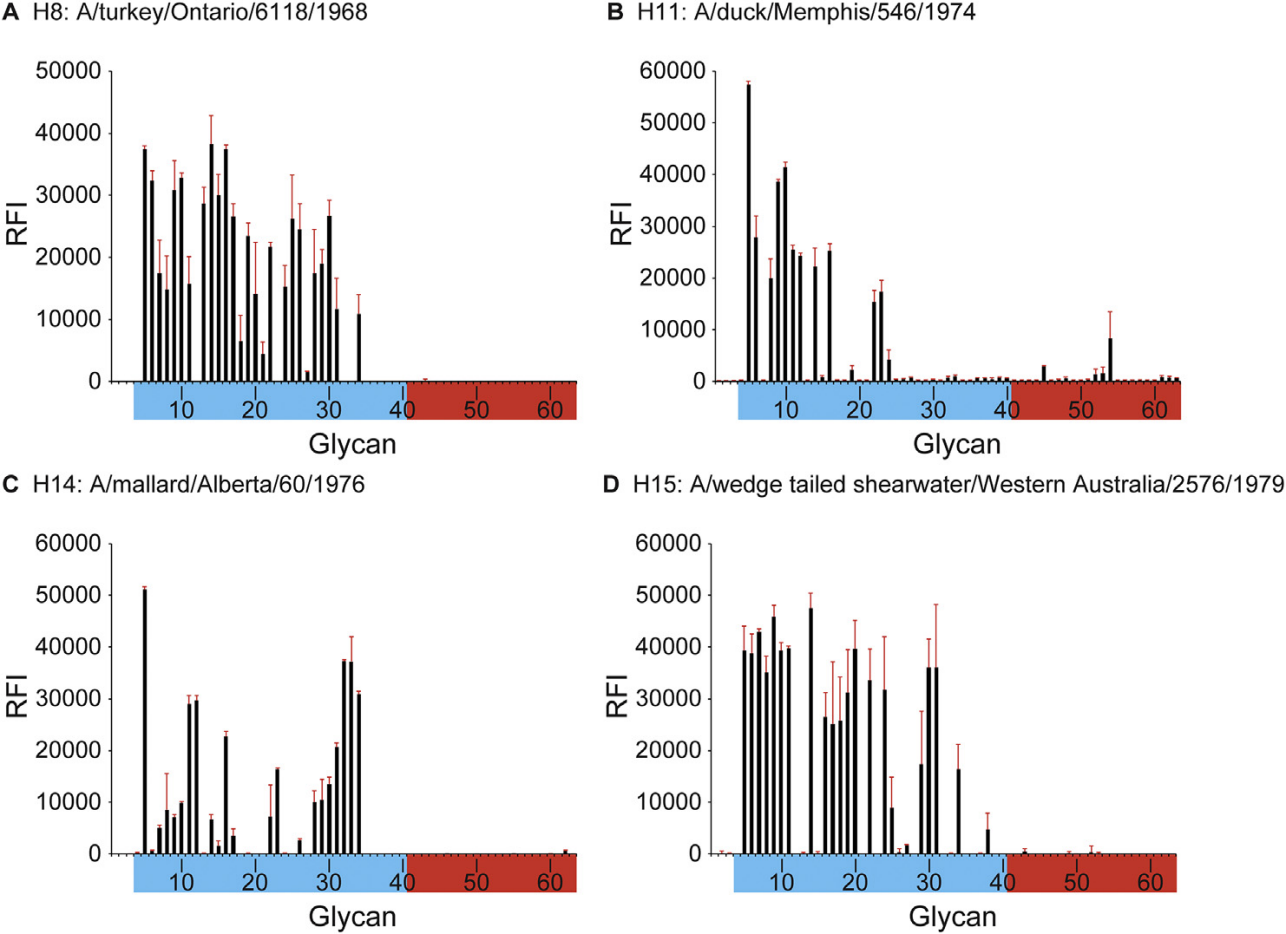
Glycan microarray analyses of avHAs. A. avH8. B. avH11. C. H14. D. avH15. Colored bars distinguish glycans that contain avian-type α2-3 sialic acid (blue) and human-type α2-6 sialic acid (red). Error bars arestandard deviations from six independent replicates on the array. Each of the numbered glycans' structures are listed in Table 6.

To improve our understanding of the interactions between these avian viruses and host receptors, glycan-binding studies of avH8, avH11, avH14 and avH15 recHAs were carried out using version 1 glycan microarrays. Results revealed a strict α2-3 linked sialic acid binding for all four avian subtype HAs (Figure 5A–D). Only avH11 recHA revealed limited binding to one α2-6 glycans (#54; Neu5Acα2-6GalNAcβ1-4GlcNAcβ) (Figure 5B).

### 2.5 Molecular characterization of swine influenza virus H4 HA

More than 2200 H4 HA sequences are available in the GISAID database and these sequenced H4 viruses have been found associated with all 9 NAs. There is also one documented example of a highly pathogenic A(H4N2) virus isolated from a quail in the U.S. (A/quail/California/D113023808/2012) (Wong et al., 2014). While distinct lineages of avian H4 viruses have been circulating globally in bird populations, at least since 1956 (Donis et al., 1989), only five sequences have been documented for the isolation of H4 influenza viruses from naturally infected pigs (Hu et al., 2012; Karasin et al., 2000; Su et al., 2012). In December 2015, an A(H4N6), A/swine/Missouri/A01727926/2015 (swH4), virus was isolated from pigs exhibiting respiratory symptoms on a farm in Missouri (Abente et al., 2017). Phylogenetic analysis revealed all the genes segments to be of avian origin. Recently an older Canadian swine H4N6 virus (A/swine/Ontario/01911_1/1999) was structurally characterized (Song et al., 2017), and structural based sequence alignment of the Canadian swH4 virus with the Missouri swH4 HA revealed seven amino acids differences. However, none of the amino acid differences are located in or near the RBS.

**Figure 6:**
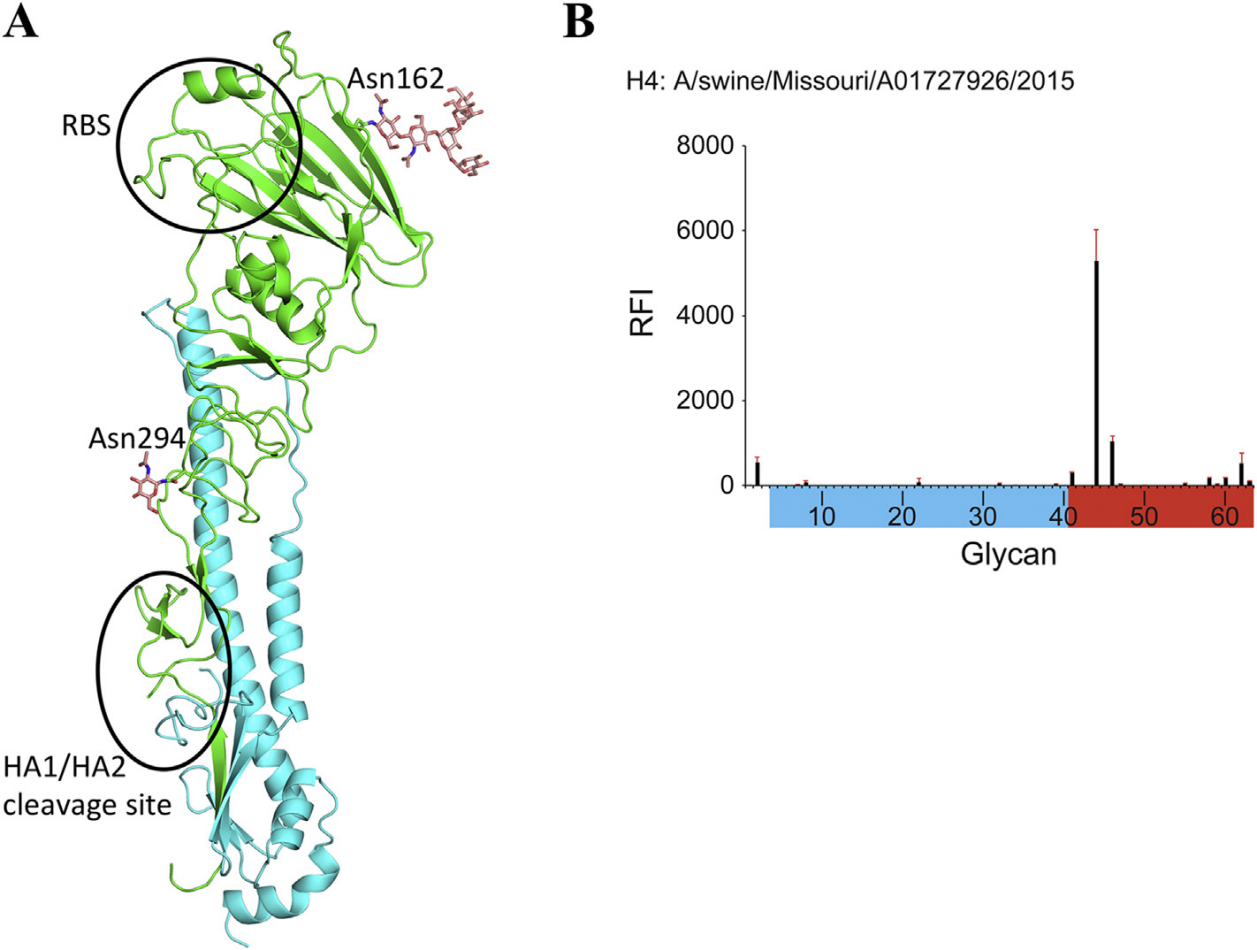
A. The overall structure of swH4 monomer, with occupied glycosylation sites shown as sticks. B. The glycan microarray analysis of swHA. Colored bars distinguish glycans that contain avian-type α2-3 SA (blue), and human-type α2-6 SA (red).

In this report, the structure of swH4 HA has been determined at 2.2 Å (Table 2). There are five predicted N-glycosylation sites, Asn2, Asn18, Asn162, and Asn294 on the HA1 and Asn154 on the HA2. Only two of these sites have interpretable electron density. Asn162 has interpreted density to build in six glycans and Asn294 has only one *N*-acetylglucosamine (GlcNAc) attached. A larger glycan was interpretable at Asn162 because this glycan makes contact with the main chain nitrogen of an adjacent chain (Trp219) within the same trimer and residues Gln122 and Asn168 in different trimers within the crystal. The overall structure of swH4 HA resembles other available HA structures (Figure 6A). With Cα atoms superimposed, the RMSDs are shown in Table 3. The RMSD of the closest related avian H4N6 (A/duck/Czechoslovakia/1956; PDB ID: 5XL1) and swine H4N6 (A/swine/Ontario/01911-1/99; PDB ID: 5XL2) are 0.36 Å and 1.03Å, respectively.

**Figure 7:**
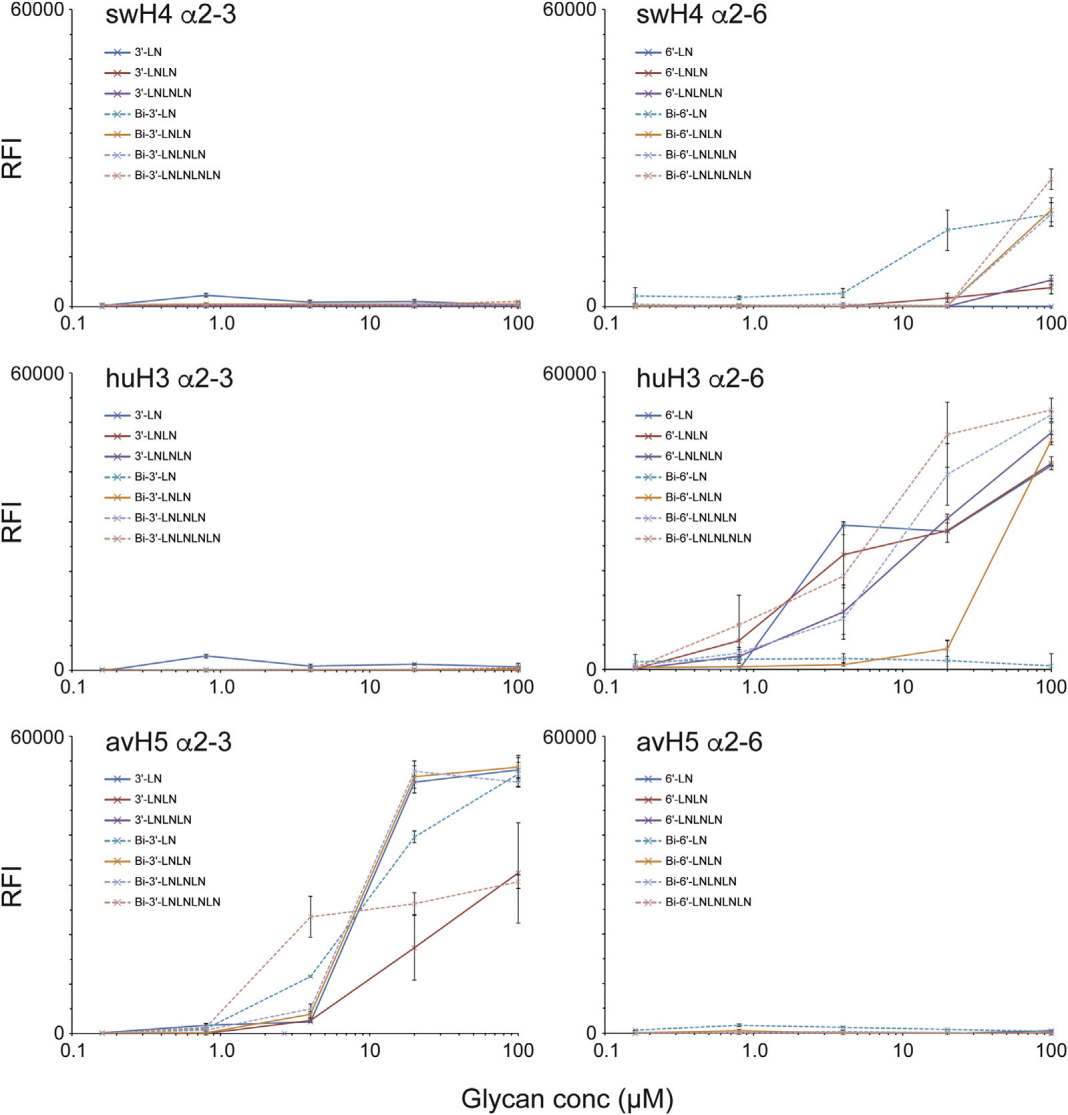
Glycan microarray analysis of swH4 HA compared to human and avian recHAs. A second glycan array containing only a limited set of glycans containing a mix of linear and biantennary α2-3 and α2-6 linked sialosides of different lengths (from 1 to 4 LacNAc repeats) and spotted onto the array at different glycan concentrations, were used to assess both HA binding specificity and avidity.

Glycan microarray analysis of swH4 with version 1 microarrays, revealed a restricted binding to α2-6 glycans, in particular the biantennary α2-6 di-LacNAc sialoside (#44; Neu5Acα2-6Galβ1-4GlcNAcβ1-3Galβ1-4GlcNAcβ1-2Manα1-3[Neu5Acα2-6Galβ1-4GlcNAcβ1-3Galβ1-4GlcNAcβ1-2Manα1-6]Manβ1-4GlcNAcβ1-4GlcNAcβ; Bi-6′-LNLN) (Figure 6B). To further look into this strict receptor specificity, we also analyzed the H4 recHA binding to a different glycan array containing α2-3 and α2-6 linear and branched glycans of different lengths and imprinted at different concentrations (version 2 microarrays). Results once again highlighted a specific, but weaker binding preference for α2-6 glycans compared to the huH3 control recHA, with no binding to the α2-3 glycans (Figure 7). The swH4 also bound better to biantennary α2-6 sialosides than the linear glycans on the array. In addition, binding was mainly detected on spots loaded with 100μM of glycan, the highest glycan concentration on the array, highlighting the weaker binding of this recHA when compared to the huH3 and avH5 controls (Figure 7).

## 3 Discussion

Hemagglutinin is the most important surface glycoprotein of influenza that enables the virus to bind to host cell receptors and gain entry into the host cell. The first three-dimensional structure of HA was published in 1981 by Wilson, Wiley and Skehel (Wilson et al., 1981). Since then, many subtypes of HA structures have been determined with only H8, H11 and H12 not yet reported in the PDB. HA is a trimer of identical subunits, each monomer consists of a globular head containing the RBS, and a stalk domain including the HA1/HA2 cleavage site. All HA RBS are composed of three structural elements (a 190-helix, a 220-loop, and a 130-loop) in addition to four highly conserved residues (Tyr98, Trp153, His183, and Tyr195) that contribute to the overall architecture of the RBS. There are some group-specific structural features of different subtype HAs, such as the extended 150-loop of H7, H10 and H15; interhelix loop variation etc., but all HAs maintain their ability to effectively bind to sialic acid receptors and mediate virus entry and fusion.

In this study, we report the first avH8 and avH11 HAs to be structurally characterized. The overall structure of these two subtypes HAs show high similarity with all other available HAs, especially the group-1 HAs. Rotation of the head domain does not seem to relate to any of the HA functions, such as receptor binding, and analysis of these rotations revealed no distinct subgroup clustering (Table 4). Similarly, the long alpha helices and the interhelix loop of the HA2 is involved in virus fusion as large conformational changes of the helices and interhelix loop occur during re-organization of the HA2 to its fusion form. The structures of different subtype HAs reveal subgroup-specific features in this region. Here, we also report avH14 and avH15 HA structures and both HAs also clustered with their subgroup partner HAs with respect to their interhelix loop alignment (Figure 3B). Further studies will be needed to assess whether these loop variations impact on the HA fusion process. An earlier study on the H7, H10 and H15 subgroup suggested these viruses shared common features that appear to increase their virulence in humans (Qi et al., 2014). Based on the structures, this group of HAs all have the two amino acid insertion at 150-loop to make an extended loop over the RBS. Furthermore, the extra H15 HA 260-loop may contribute to the antigenic variation of this subtype HA (Figure 2D).

Glycan microarray technology is a very useful tool to study HA binding specificities. The results of avian recHAs used in this study show that all avHAs have strict avian-like receptor binding preferences. Swine are suggested to be a genetic mixing vessel due to the frequent co-infection and recombination of influenza viruses from various sources and lineages (Ma et al., 2008). Influenza infection of pigs is an important turning point in the evolution and ecology of IAVs due to the dual susceptibility of pigs to human and avian influenza viruses (Ma et al., 2008). We performed molecular characterization of the HA from the swH4 virus isolated from Missouri in December 2015. The overall structure of swH4 HA is still similar to other subtype HAs. However, interestingly, the RBS of swH4 HA has Leu223 and Ser225 constellation (Leu226 and Ser228 in H3 numbering), which are recognized as the key residues responsible for AIVs adaption to human influenza virus for H2 and H3 subtypes (Connor et al., 1994; Rogers et al., 1985). Among 2228 H4 sequences in GISAID, only 3 sequences possess the Leu223/Ser225 constellation, while 3 have the Gln223Ser225 constellation (Table 5). Glycan binding results reported here are in line with what was observed previously with studies on an older swine H4 virus, A/swine/Ontario/01911/1999, with the same Leu223/Ser225 constellation, although the assays employed here were different (Song et al., 2017).

**Table 5:** Numbers of virus isolates with different key residue combinations in the RBS of all H4 HA.

H4 Receptor binding site residues				Number of virus isolates 2228 in total
187(190)∗	222(225)	223(226)	225(228)	
E	S	Q	G	8
E	D	Q	G	1
E	E	Q	G	1
E	G	Q	G	2207 Consensus
E	G	L	S	3#
E	G	Q	S	3$
E	G	Q	A	5

∗ H3 numbering in parenthesis.

# EPI8131: A/Swine/Ontario/01911-1/99 EPI8127: A/Swine/Ontario/01911-2/99 EPI718518: A/swine/Missouri/A01727926/2015.

$ EPI1177844: A/duck/Shiga/37/2007 EPI69006: A/environment/Maryland/1101/2006 EPI453348: A/northern pintail/Interior Alaska/10BM07242R0/2010.

The requirements for virus host switching are complicated and changing receptor binding specificity is not the only major barrier for adaptation of avian influenza viruses to humans. With swine as a possible intermediate host, the swH4 virus HA has the Leu223 and Ser225 constellation and thus the H4 virus has the potential to adapt further or reassort with human viruses in pigs.

## 4 Conclusions

In this study, we presented structural and glycan binding data of four avHAs and one swHA. While all the HAs have a similar overall structure, they also maintain group-specific structural features of different subtype HAs, such as an extended 150-loop for the H15 and similar interhelix loop variations. Although the avHAs revealed strict receptor binding preferences, the swine H4 however showed markedly reduced binding to avian-type receptors and weak binding to human receptors. While this virus may not yet be considered a virus with pandemic potential, the H4 subtype should be included in the list of viruses to continue watching. With the growing number of human and zoonotic infections by AIVs, it raises the public health concern that one or more of these viruses will eventually evolve into a variant capable of causing another global pandemic. Continued surveillance of domestic and wild bird populations as well as domestic animals is critical. Moreover, the transmission of AIVs to swine should be a warning sign, and these types of incidents should be monitored and investigated in detail.

## 5 Materials and methods

### 5.1 Recombinant HA cloning

cDNAs encoding the mature ectodomains of each HA (listed in Table 1) were synthesized as codon optimized constructs (Genscript Inc.). Genes were subcloned into the pAcGP67-B baculovirus transfer vector, in frame with an N-terminal baculovirus GP67 signal peptide, a C-terminal thrombin cleavage site, a T4 fibritin trimerization tag for generating functional trimers, and a His-Tag for purification (Stevens et al., 2004). Transfection was carried out utilizing baculovirus vector DNA and transfection reagents from AB Vector (San Diego, CA) using their suggested transfection protocol. Virus amplification was performed in *Sf*9 insect cells.

### 5.2 HA expression and purification

Large scale protein expression was performed in High Five™ insect cells. Secreted soluble trimeric recHA protein was recovered from the cell culture supernatant by tangential flow filtration through a 30kDa molecular weight cut-off membrane, metal affinity chromatography and gel filtration chromatography. For structural studies, trimeric recHA proteins were subjected to trypsin digestion (1:1000 w/w ratio of trypsin:protein). For crystallization trials, HAs were concentrated to 13-17 mg/mL during buffer exchanging into 10 mM Tris-HCl, 50 mM NaCl, pH 8.0.

### 5.3 HA crystallization

Either a Topaz™ Free Interface Diffusion (FID) Crystallizer system (Fluidigm Corporation, San Francisco, CA) or a Formulatrix NT8 (Formulatrix, Inc., Bedford, MA) were used to set up the initial crystallization screens. Conditions in which crystals were observed were optimized using a modified method for microbatch under oil (Chayen, 2007) at 20 °C. Crystals were mounted onto pins, flash-cooled at 100 K, and datasets were collected at the Argonne National Laboratory Advanced Photon Source (APS) beamline 22-ID or 22-BM, and processed with the SACLEPACK suite (Otwinowski and Minor, 1997). Detailed information on the crystallization conditions for each HA is included in Table 2.

### 5.4 HA structure refinement

HA structures were solved by molecular replacement with Phaser (McCoy et al., 2007). Initial models were built with their correct sequences by Coot (Emsley et al., 2010), and refined with Phenix (Adams et al., 2010) and REFMAC using TLS refinement (Winn et al., 2001). The final models were assessed using MolProbity (Davis et al., 2007). Data processing and refinement statistics are shown in Table 2. Unless specified otherwise all residue numbering is based on the mature HA protein. For structural analyses, PDBs of other subtype HAs were chosen based on the latest deposition date, resolution of the structure, or if they are the only available example in PDB.

### 5.5 Glycan binding analyses

RecHA glycan microarray analyses have been described previously (Yang et al., 2013). Briefly, HA-antibody precomplexes were prepared in a molar ratio of 4:2:1 and mixtures were incubated on ice (60 min), followed by dilution with 500 μl phosphate-buffered saline (PBS) buffer containing 2% (wt/vol) bovine serum albumin and streptavidin-Alexa Fluor 488 (1:1,000 [vol/vol]; Life Technologies). Mixtures were applied to the microarray slides and incubated on ice for an additional 60 min. Slides were then sequentially washed in PBS with 0.05% Tween 20 (vol/vol), PBS, and deionized water and then dried. Slides were scanned using an Innoscan 100 microarray scanner (Innopsys Inc.), and images were analyzed using ImaGene version 9 software (BioDiscovery Inc.). Table 6 lists the specific glycans on the large version 1 array.

**Table 6:** Glycans present on version 1 microarrays.

Glycan No.	Structure
1	Neu5Acα
2	Neu5Acα
3	Neu5Acβ
4	Neu5Acα2-3(6-O-Su)Galβ1-4GlcNAcβ
5	Neu5Acα2-3Galβ1-3[6OSO3]GalNAcα
6	Neu5Acα2-3Galβ1-4[6OSO3]GlcNAcβ
7	Neu5Acα2-3Galβ1-4(Fucα1-3)[6OSO3]GlcNAcβ
8	Neu5Acα2-3Galβ1-3[6OSO3]GlcNAcβ
9	Neu5Acα2-3Galβ1-3(Neu5Acα2-3Galβ1-4)GlcNAcβ
10	Neu5Acα2-3Galβ1-3(Neu5Acα2-3Galβ1-4GlcNAcβ1-6)GalNAcα
11	Neu5Acα2-3Galβ1-4GlcNAcβ1-2Manα1-3(Neu5Acα2-3Galβ1-4GlcNAcβ1-2Manα1-6)Manβ1-4GlcNAcβ1-4GlcNAcβ
12	Neu5Acα(2-3)-Galβ(1-4)-GlcNAcβ(1-3)-Galβ(1-4)-GlcNAcβ(1-2)-Manα(1-3)-[Neu5Acα(2-3)-Galβ(1-4)-GlcNAcβ(1-3)-Galβ(1-4)-GlcNAcβ(1-2)-Manα(1-6)]-Manβ(1-4)-GlcNAcβ(1-4)-GlcNAcβ
13	Neu5Acα2-3Galβ
14	Neu5Acα2-3Galβ1-3GalNAcα
15	Neu5Acα2-3Galβ1-3GlcNAcβ
16	Neu5Acα2-3Galβ1-3GlcNAcβ
17	Neu5Acα2-3Galβ1-4Glcβ
18	Neu5Acα2-3Galβ1-4Glcβ
19	Neu5Acα2-3Galβ1-4GlcNAcβ
20	Neu5Acα2-3Galβ1-4GlcNAcβ
21	Neu5Acα2-3GalNAcβ1-4GlcNAcβ
22	Neu5Acα2-3Galβ1-4GlcNAcβ1-3Galβ1-4GlcNAcβ
23	Neu5Aca2-3Galβ1-3GlcNAcβ1-3Galβ1-4GlcNAcβ
24	Neu5Acα2-3Galβ1-4GlcNAcβ1-3Galβ1-4GlcNAcβ1-3Galβ1-4GlcNAcβ
25	Neu5Acα2-3Galβ1-4GlcNAcβ1-3Galβ1-3GlcNAcβ
26	Neu5Acα2-3Galβ1-3GalNAcα
27	Galβ1-3(Neu5Acα2-3Galβ1-4(Fucα1-3)GlcNAcβ1-6)GalNAcα
28	Neu5Acα2-3Galβ1-3(Fucα1-4)GlcNAcβ
29	Neu5Acα2-3Galβ1-4(Fucα1-3)GlcNAcβ
30	Neu5Acα2-3Galβ1-4(Fucα1-3)GlcNAcβ
31	Neu5Acα2-3Galβ1-4(Fucα1-3)GlcNAcβ1-3Galβ
32	Neu5Acα2-3Galβ1-3[Fucα1-4]GlcNAcβ1-3Galβ1-4[Fucα1-3]GlcNAcβ
33	Neu5Acα2-3Galβ1-3[Fucα1-3]GlcNAcβ1-3Galβ1-4[Fucα1-3]GlcNAcβ
34	Neu5Acα2-3Galβ1-4(Fucα1-3)GlcNAcβ1-3Galβ1-4(Fucα1-3)GlcNAcβ1-3Galβ1-4(Fucα1-3)GlcNAcβ
35	Neu5Acα2-3(GalNAcβ1-4)Galβ1-4GlcNAcβ
36	Neu5Acα2-3(GalNAcβ1-4)Galβ1-4GlcNAcβ
37	Neu5Acα2-3(GalNAcβ1-4)Galβ1-4Glcβ
38	Galβ1-3GalNAcβ1-4(Neu5Acα2-3)Galβ1-4Glcβ
39	Fucα1-2Galβ1-3GalNAcβ1-4(Neu5Acα2-3)Galβ1-4Glcβ
40	Fucα1-2Galβ1-3GalNAcβ1-4(Neu5Acα2-3)Galβ1-4Glcβ
41	Neu5Acα2-6Galβ1-4[6OSO3]GlcNAcβ
42	Neu5Acα2-6Galβ1-4GlcNAcβ1-2Manα1-3(Galβ1-4GlcNAcβ1-2Manα1-6)Manβ1-4GlcNAcβ1-4GlcNAcβ
43	Neu5Acα2-6Galβ1-4GlcNAcβ1-2Manα1-3(Neu5Acα2-6Galβ1-4GlcNAcβ1-2Manα1-6)Manβ1-4GlcNAcβ1-4GlcNAcβ
44	Neu5Acα2-6Galβ1-4GlcNAcβ1-3Galβ1-4GlcNAcβ1-2Manα1-3[Neu5Acα2-6Galβ1-4GlcNAcβ1-3Galβ1-4GlcNAcβ1-2Manα1-6]Manβ1-4GlcNAcβ1-4GlcNAcβ
45	Neu5Acα2-6Galβ1-4GlcNAcβ1-3Galβ1-4GlcNAcβ1-3Galβ1-4GlcNAcβ1-2Manα1-3[Neu5Acα2-6Galβ1-4GlcNAcβ1-3Galβ1-4GlcNAcβ1-3Galβ1-4GlcNAcβ1-2Manα1-6]-Manβ1-4GlcNAcβ1-4GlcNAcβ
46	Neu5Acα2-6Galβ1-4GlcNAcβ1-3Galβ1-4GlcNAcβ1-3[Neu5Acα2-6Galβ1-4GlcNAcβ1-3Galβ1-4GlcNAcβ1-6]GalNAca
47	Neu5Acα2-6Galβ1-4GlcNAcβ1-3[Neu5Acα2-6Galβ1-4GlcNAcβ1-6]GalNAca
48	Neu5Acα2-6GalNAcα
49	Neu5Acα2-6Galβ
50	Neu5Acα2-6Galβ1-4Glcβ
51	Neu5Acα2-6Galβ1-4Glcβ
52	Neu5Acα2-6Galβ1-4GlcNAcβ
53	Neu5Acα2-6Galβ1-4GlcNAcβ
54	Neu5Acα2-6GalNAcβ1-4GlcNAcβ
55	Neu5Acα2-6Galβ1-4GlcNAcβ1-3GalNAcα
56	Neu5Acα2-6Galβ1-4GlcNAcβ1-3Galβ1-4GlcNAcβ
57	Neu5Acα2-6Galβ1-4GlcNAcβ1-3Galβ1-4GlcNAcβ1-3GalNAcα
58	Neu5Aca2-6Galβ1-4GlcNAcβ1-3Galβ1-4GlcNAcβ1-3Galβ1-4GlcNAcβ
59	Neu5Acα2-6Galβ1-4GlcNAcβ1-3Galβ1-4(Fucα1-3)GlcNAcβ1-3Galβ1-4(Fucα1-3)GlcNAcβ
60	Galβ1-3(Neu5Acα2-6)GlcNAcβ1-4Galβ1-4Glcβ-Sp10
61	Neu5Acα2-6[Galβ1-3]GalNAca
62	Neu5Acα2-6Galβ1-4GlcNAcβ1-6[Galβ1-3]GalNAca
63	Neu5Acα2-6Galβ1-4GlcNAcβ1-3Galβ1-4GlcNAcβ1-6[Galβ1-3]GalNAca

A second array (version 2) containing only a limited set of glycans was also used for analyzing the H4 recHA. These glycans, a mix of linear and biantennary α2-3 and α2-6 linked sialosides of different lengths (from 1 to 4 LacNAc repeats), were imprinted on the array at different concentrations (100μM, 20μM, 4μM, 0.8μM and 0.16μM) to assess both specificity and avidity. This alternate glycan array slide was processed using the same procedure as described above. Table 7 lists the glycans on the version 2 array.

**Table 7:** Glycans present on version 2 microarrays.

Glycan No.	Structure
3′-LN	Neu5Acα2-3Galβ1-4GlcNAcβ
3′-LNLN	Neu5Acα2-3Galβ1-4GlcNAcβ1-3Galβ1-4GlcNAcβ
3′-LNLNLN	Neu5Acα2-3Galβ1-4GlcNAcβ1-3Galβ1-4GlcNAcβ1-3Galβ1-4GlcNAcβ
Bi-3′-LN	Neu5Acα2-3Galβ1-4GlcNAcβ1-2Manα1-3[Neu5Acα2-3Galβ1-4GlcNAcβ1-2Manα1-6]-Manβ1-4GlcNAcβ1-4GlcNAcβ
Bi-3′-LNLN	Neu5Acα2-3-Galβ1-4-GlcNAcβ1-3Galβ1-4GlcNAcβ1-2Manα1-3[Neu5Acα2-3Galβ1-4GlcNAcβ1-3Galβ1-4GlcNAcβ1-2Manα1-6]-Manβ1-4GlcNAcβ1-4GlcNAcβ
Bi-3′-LNLNLN	Neu5Acα2-3Galβ1-4GlcNAcβ1-3Galβ1-4GlcNAcβ1-3Galβ1-4GlcNAcβ1-2Manα1-3[Neu5Acα2-3Galβ1-4GlcNAcβ1-3Galβ1-4GlcNAcβ1-3Galβ1-4GlcNAcβ1-2Manα1-6]-Manβ1-4GlcNAcβ1-4GlcNAcβ
Bi-3′-LNLNLNLN	Neu5Acα2-3Galβ1-4GlcNAcβ1-3Galβ1-4GlcNAcβ1-3Galβ1-4GlcNAcβ1-3Galβ1-4GlcNAcβ1-2Manα1-3[Neu5Acα2-3Galβ1-4GlcNAcβ1-3Galβ1-4GlcNAcβ1-3Galβ1-4GlcNAcβ1-3Galβ1-4)GlcNAcβ1-2Manα1-6]-Manβ1-4GlcNAcβ1-4GlcNAcβ
6′-LN	Neu5Acα2-6Galβ1-4GlcNAcβ
6′-LNLN	Neu5Aca2-6Galβ1-4GlcNAcβ1-3Galβ1-4GlcNAcβ
6′-LNLNLN	Neu5Aca2-6Galβ1-4GlcNAcβ1-3Galβ1-4GlcNAcβ1-3Galβ1-4GlcNAcβ
Bi-6′-LN	Neu5Acα2-6Galβ1-4GlcNAcβ1-2Manα1-3[Neu5Acα2-6Galβ1-4GlcNAcβ1-2Manα1-6]-Manβ1-4GlcNAcβ1-4GlcNAcβ
Bi-6′-LNLN	Neu5Acα2-6Galβ1-4GlcNAcβ1-3Galβ1-4GlcNAcβ1-2Manα1-3[Neu5Acα2-6Galβ1-4GlcNAcβ1-3Galβ1-4GlcNAcβ1-2Manα1-6]Manβ1-4GlcNAcβ1-4GlcNAcβ
Bi-6′-LNLNLN	Neu5Acα2-6Galβ1-4GlcNAcβ1-3Galβ1-4GlcNAcβ1-3Galβ1-4GlcNAcβ1-2Manα1-3[Neu5Acα2-6Galβ1-4GlcNAcβ1-3Galβ1-4GlcNAcβ1-3Galβ1-4GlcNAcβ1-2Manα1-6]-Manβ1-4GlcNAcβ1-4GlcNAcβ
Bi-6′-LNLNLNLN	Neu5Acα2-6 Galβ1-4GlcNAcβ1-3Galβ1-4GlcNAcβ1-3Galβ1-4GlcNAcβ1-3Galβ1-4GlcNAcβ1-2Manα1-3[Neu5Acα2-6Galβ1-4GlcNAcβ1-3Galβ1-4GlcNAcβ1-3Galβ1-4GlcNAcβ1-3Galβ1-4GlcNAcβ1-2Manα1-6]-Manβ1-4GlcNAcβ1-4GlcNAcβ

### 5.6 PDB accession codes

All HA models with their atomic coordinates and structure factors are available from the RCSB PDB database (www.pdb.org) under the accession codes listed in Table 2.

## Declarations

### Author contribution statement

Hua Yang, Paul J. Carney, Jessie C. Chang: Conceived and designed the experiments; Performed the experiments; Analyzed and interpreted the data; Wrote the paper.

James Stevens: Conceived and designed the experiments; Analyzed and interpreted the data; Wrote the paper.

### Funding statement

This work was supported by the 10.13039/100000030Centers for Disease Control and Prevention.

### Competing interest statement

The authors declare no conflict of interest.

### Additional information

No additional information is available for this paper.
